# Analyse de la gestion et des aspects épidémiologiques de l’épidémie COVID-19 au Sénégal à un an d’évolution

**DOI:** 10.11604/pamj.2023.46.5.30693

**Published:** 2023-09-06

**Authors:** Abdoulaye Bousso, Ibrahima Sonko, Aissatou Lakhe, Alioune Badara Ly, Papa Samba Ba, Allé Baba Dieng, Sadiya Aïdara, Madeleine Khady Sarr, Ndiouma Faye

**Affiliations:** 1 Centre des Opérations d’Urgence Sanitaire, Ministère de la Santé, Dakar, Sénégal,; 2 Service des Maladies Infectieuses et Tropicales, CHU de Fann, Ministère de la Santé, Dakar, Sénégal,; 3 Service des Maladies Infectieuses, Hôpital Principal de Dakar, Sénégal,; 4 Medicines, Technologies and Pharmaceutical Services Program, Dakar, Sénégal,; 5 Agence Nationale pour la Statistique et la Démographie, Dakar, Sénégal

**Keywords:** COVID-19, Sénégal, épidémiologie, COVID-19, Senegal, epidemiology

## Abstract

**Introduction:**

après une année d´évolution, la pandémie COVID-19 continue d´être un fardeau pour le monde. Le continent Africain, malgré qu´il ne soit pas touché comme on le pensait souffre des conséquences sanitaires et économiques. Le Sénégal, à l´instar des autres pays africains continue de faire face à cette pandémie. L´objectif de ce travail est d´analyser la stratégie de gestion et le profil épidémiologique de l´épidémie au Sénégal après un an d´évolution.

**Méthodes:**

nous avons répertorié tous les patients testés COVID-19 positifs par RT-PCR au cours de la première année de l´épidémie, du 2 mars 2020 (date du premier cas) au 1^er^ mars 2021 sur l'ensemble du territoire national. Une analyse des données épidémiologiques a été réalisée.

**Résultats:**

il a été diagnostiqué 34 732 cas positifs, enregistrés en une année, avec un taux de létalité à 2,5%. Toutes les régions administratives du pays sont touchées, avec Dakar, la capitale, comme l'épicentre de l´épidémie. La prédominance masculine est notée sur le nombre de cas positifs et de décès. La moyenne d´âge était de 47 ans et le taux de guérison était de 83,5%. Les personnes de plus de 60 ans étaient les plus vulnérables, en particulier avec des comorbidités cardiovasculaires.

**Conclusion:**

la stratégie du Sénégal dans la gestion de la COVID-19 a été soulignée sur le plan international. Elle a été dynamique, tirant des expériences de la gestion antérieure d´évènements de santé publique comme Ebola. La pandémie du COVID-19 a mis à rude épreuve nos systèmes de santé fragiles. Cependant, la réaction et les résultats obtenus mettent en évidence les progrès importants réalisés par notre pays, contribuant à assurer la résilience du système de santé.

## Abstract (en)

**Introduction:**

after a year of evolution, the COVID-19 pandemic continues to be a burden. The African continent has not had the high case and death rates from COVID-19 as was predicted early in the pandemic. However, coronavirus is placing severe strains on Africa´s health and economic sectors. Senegal, like other African countries, continues to face this pandemic. The purpose of this study is to evaluate the management strategy and the epidemiological profile of the pandemic in Senegal one year on.

**Methods:**

we listed all patients who tested positive for COVID-19 by RT-PCR throughout the country during the first year of the pandemic, from 2 March 2020 (first case date) to 1^st^ March 2021. Epidemiological data were analysed.

**Results:**

a total of 34,732 positive cases were diagnosed and recorded in one year, with a case-fatality rate of 2.5%. All the country´s administrative regions had been affected. Dakar, the capital, was the epicentre of the pandemic. Male predominance was established on the basis of the number of positive cases and deaths. The average age of subjects was 47 years and the cure rate was 83.5%. People over 60 were the most vulnerable, particularly those with cardiovascular co-morbidities.

**Conclusion:**

Senegal´s strategy for managing COVID-19 has received international recognition. It has been dynamic, drawing on experience from previous management of public health events such as Ebola. The COVID-19 pandemic has put a strain on our fragile health systems. However, the response and the results achieved highlight the significant progress made by our country, helping to ensure the resilience of the health system.

## Introduction

L´épidémie du nouveau coronavirus de 2019 (2019-nCoV), qui s´est déclaré à Wuhan en Chine [1,2] continue de s´étendre dans le monde et met à l´épreuve les systèmes de santé [3]. Elle est rapidement devenue urgence de santé publique de portée internationale [4], touchant tous les continents [5]. Après l´avoir baptisée COVID-19 [6], l´OMS l´a déclarée comme pandémie [7]. Le 2 mars 2020, le Sénégal a confirmé son premier importé de la France. Pendant la première année d´évolution, l´épidémie a évolué en deux vagues. Le présent travail se propose de faire une analyse de l´évolution épidémiologique de la maladie au Sénégal ainsi que des stratégies de réponse mises en œuvre.

## Méthodes

**Site d´étude:** le Sénégal est le pays le plus à l´ouest, sur la façade atlantique du continent africain, Il est frontalier avec 5 pays: le Mali à l´Est, la Mauritanie au Nord, les Républiques de Guinée et Guinée Bissau au Sud et la Gambie enclavée dans le centre Sénégal. Il s´étend sur une superficie de 196 722 km^2^ et est divisé en 14 régions administratives. La population est estimée à 16 209 125 habitants en 2019 [8] dont les 23% sont concentrés à Dakar, la capitale du pays. Le système de santé du Sénégal est organisé sur un mode pyramidal. Le pays est divisé en 14 régions médicales superposées aux régions administratives. Chaque région médicale est divisée en districts sanitaires, qui sont au nombre de 79. Le Sénégal compte 35 hôpitaux publics, 102 centres de santé et 1415 postes de santé [9]. Des sites de traitement dédiés à la prise en charge des cas COVID-19 confirmés ont été progressivement mis en place dans l´ensemble du pays.

**Période d´étude:** elle couvre une période d´une année, du 02 mars 2020, date de confirmation du premier cas, au 1^er^ mars 2021.

**Population d´étude:** l´étude porte sur tous les cas testés positifs par la méthode RT-PCR répertoriés durant la période sur tout le pays.

**Capacité des laboratoires:** la capacité de test COVID-19 a progressivement évolué au Sénégal, avec au début un seul laboratoire capable de faire le diagnostic: l´Institut Pasteur de Dakar (IPD). Ensuite l´Institut de Recherche En Santé de Surveillance Épidémiologique et de Formation (IRESSEF) et sept autres laboratoires ont pu développer leur capacité diagnostique (Hôpital Le Dantec, Hôpital militaire de Ouakam, Laboratoire national de santé publique, Hôpital Principal de Dakar, Hôpital de Fann, Hôpital Dalal Jamm et le Centre médical interarmées du camp Lemonnier). L´institut Pasteur a appuyé la décentralisation des test PCR dans 9 régions. Ainsi 10 des 14 régions du Sénégal avaient la capacité de faire un test PCR. Pour les quatre régions sans capacité diagnostique, cela a été une décision volontaire pour rationaliser les ressources. Ces régions étant très proches des régions dotées de laboratoire habilités.

**Prise en charge:** la stratégie de prise en charge des patients COVID-19 a été dynamique. Au commencement, tous les cas positifs étaient pris en charge dans les centres de traitements dédiés. Des centres spécifiques à la prise en charge des malades ont été mis en place dans toutes les 14 régions administratives du pays. Par la suite, devant l´augmentation et l´extension géographique des cas positifs, des sites extrahospitaliers ont été aménagés. Il s´agissait d´hôtels, de centres d´accueil, de stades, etc. Ainsi, 36 sites de prise en charge ont été mises en place sur l´ensemble du pays: 27 hospitaliers et 9 extrahospitaliers. Le Gouvernement du Sénégal a très tôt débloqué des ressources propres pour endiguer la pandémie d´abord en finançant le plan de contingence élaboré par le Ministère de la Santé pour un montant de 2,409,000 $. Il a aussi pris des décisions fortes allant dans le sens de limiter les rassemblements dès le 14 mars 2020. Ainsi, l´Etat a décidé pour des durées variables: l´interdiction des manifestations publiques, la suspension des enseignements dans les écoles et universités, la fermeture des frontières et l´instauration de l´État d´urgence et d´un couvre-feu de 20h à 6h du matin.

## Résultats

**Figure 1:**
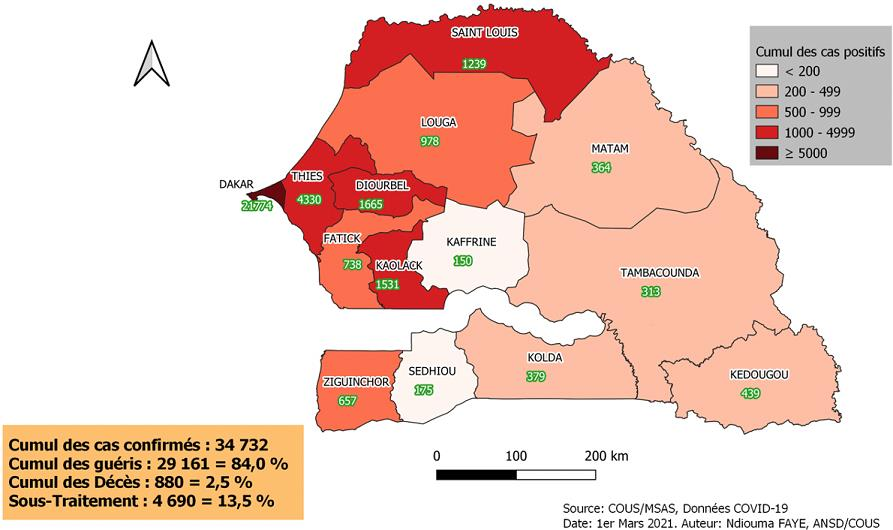
répartition des cas COVID-19 par région

**Figure 2:**
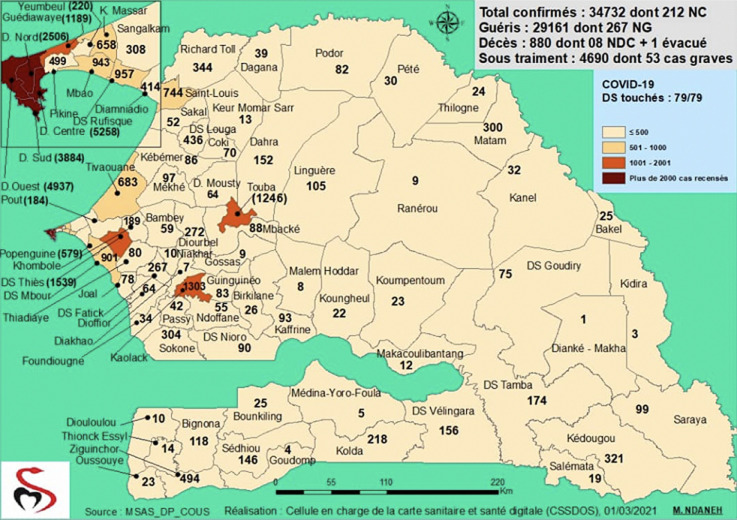
répartition des cas COVID-19 par district sanitaire

**Figure 3:**
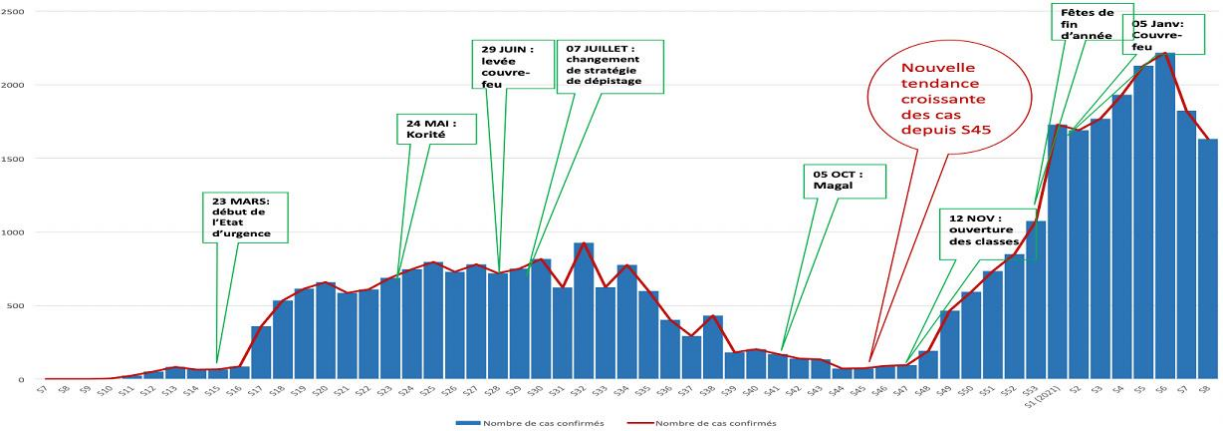
courbe épidémique hebdomadaire des cas confirmés de la COVID-19 au Sénégal

**Nombre de cas et répartition:** à date du 1^er^ mars 2021, 34 732 cas de COVID-19 ont été répertoriés et répartis sur les 14 régions, celles de Dakar, Thiès, Diourbel et Kaolack étant les plus touchés (Figure 1). Tous les 79 districts sanitaires sont touchés, soit 100 % (Figure 2). Tous les cas ont été confirmés par un test RT PCR positif. L´évolution des cas s´est faite en deux vagues: une première vague allant de la semaine 9 à la semaine 44 (pic journalier de 207 cas) et une deuxième vague à partir de la semaine 45 (pic journalier de 462 cas) (Figure 3). Les cas positifs ont été répartis en trois groupes: cas importés (infection décelée chez un voyageur entrant au Sénégal), cas contacts (infection au décours d´une contamination par une personne positive sur le territoire national) et cas communautaire (source de contamination non identifiée). Parmi les 34 732 cas confirmés, 17 705 sont des cas contacts, 16 433 cas issus de la transmission communautaires et 594 cas importés. Le premier cas de transmission communautaire a été enregistré le 19 mars 2020 dans la région de Thiès.

**Âge et sexe:** une prédominance masculine a été observée, avec une proportion de 55,5% d´hommes et 44,5% de femmes (Tableau 1). La moyenne d´âge étant de 47 ans avec des extrêmes de 7 mois et 101 ans.

**Tableau 1:** répartition par âge et sexe de la population atteinte de COVID-19 au Sénégal

Tranche âge	Sexe Masculin	Sexe Féminin	Total	Proportion
0-4 ans	182	129	311	0,9%
5-14 ans	457	416	873	2,5%
15-24 ans	1969	2066	4035	11,6%
25-34 ans	3633	3583	7216	20,8%
35-44 ans	3696	3103	6799	19,6%
45-59 ans	4098	3085	7183	20,7%
60 ans et +	5227	3088	8315	23,9%
**Total**	19262	15470	34732	-

**Figure 4:**
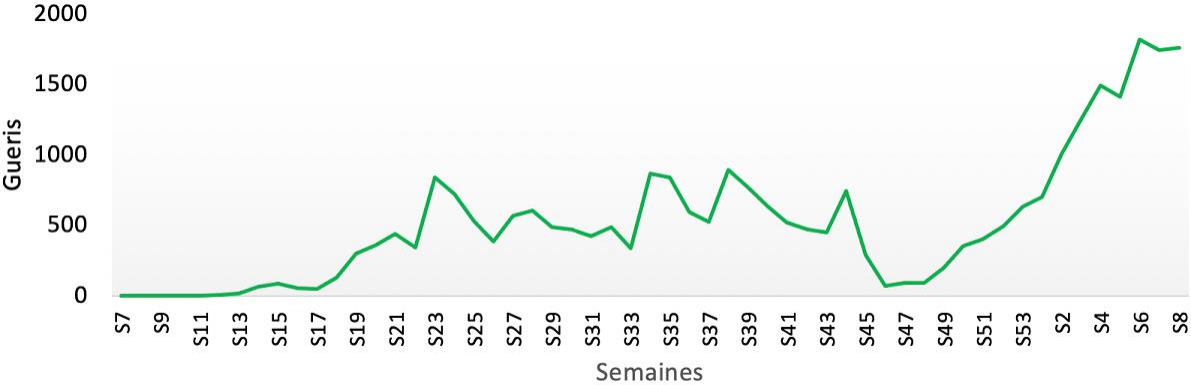
évolution hebdomadaire du nombre de guéris de la COVID-19 au Sénégal

**Taux de guérison:** à date du 1^er^ mars 2021, 29161 patients ont été déclarés guéris, avec un taux de guérison de 83,5% (Figure 4). Les patients sont déclarés guéris suite à 2 tests RT-PCR négatifs à 48 heures d´intervalle.

**Figure 5:**
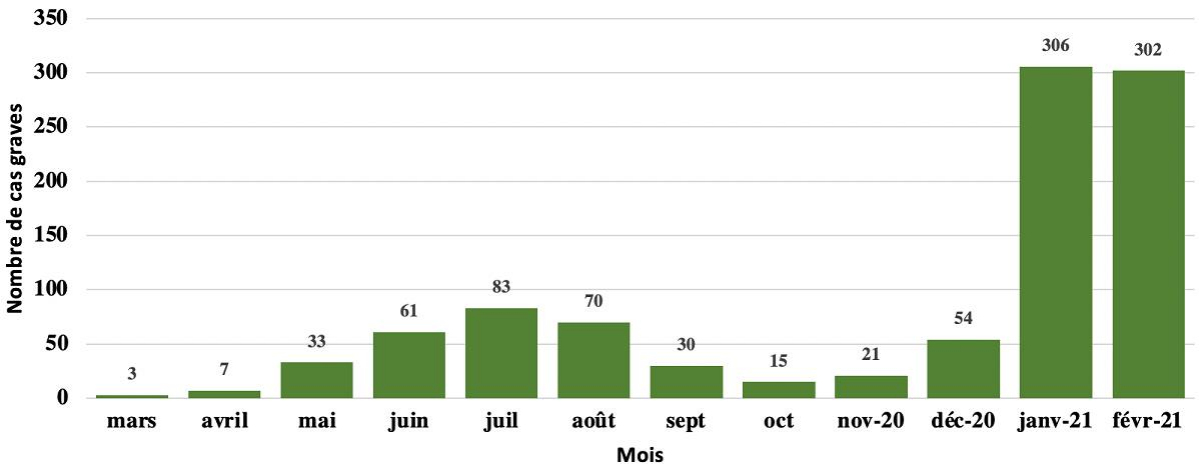
évolution mensuelle des cas graves de COVID-19 au Sénégal

**Cas graves:** au total, 985 cas graves ont été enregistrés entre le 2 mars 2020 et le 1^er^ mars 2021 avec un pic de 306 au mois de janvier 2021 (Figure 5).

**Figure 6:**
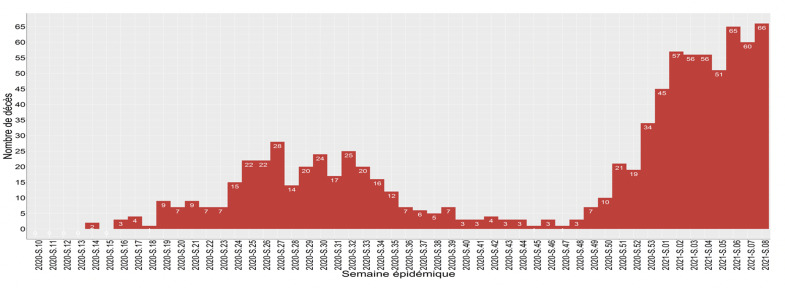
évolution hebdomadaire des décès de la COVID-19 au Sénégal

**Mortalité:** à un an d´épidémie, 880 patients sont décédés (Figure 6). La moyenne d´âge de ces derniers est de 69 ans avec des extrêmes de 3 à 101 ans. Le taux de létalité était de 2,5%. La majorité des décès est masculine avec 593 hommes contre 287 femmes décédées (Tableau 2). Les décès ont été répertoriés sur trois sites : les centre des traitements spécialisés, les structures de santé et les domiciles (Tableau 3). La plupart des personnes décédées avaient une comorbidité (69,5%). Les pathologies cardiovasculaires et métaboliques arrivaient en tête (Tableau 4).

**Tableau 2:** répartition des patients décédés de COVID-19 au Sénégal par tranche âge et par sexe

Tranche d´âge	Sexe Masculin	Sexe Féminin	Total	Proportion (%)
0-4 ans	2	0	2	0,2
5-14 ans	0	1	1	0,1
15-24 ans	3	2	5	0,6
25-34 ans	10	16	26	2,9
35-44 ans	31	24	55	6,3
45-59 ans	96	44	140	16
60 ans et +	451	200	651	73,9
**Total**	593	287	880	-

**Tableau 3:** répartition des décès de COVID-19 au Sénégal par lieu

Lieu du décès	Effectif	Proportion (%)
Centre de traitement spécialisé	778	88,4
Structure de santé	44	5
Domicile	58	6,6
**Total**	880	100

**Tableau 4:** répartition des comorbidités présentes chez les patients décédés de COVID-19

Type de comorbidité	Effectif	Proportion
HTA	202	33%
Diabète	177	29%
Accident vasculaire cérébral	57	9,3%
Obésité	48	7,8%
Cardiopathie	59	9,6%
Maladie rénale	23	3,8%
Asthme	19	3,1%
Bronchopneumopathie chronique obstructive (BPCO)	10	1,6%
Autres	17	2,7%
**Total**	612	100%

**Alertes reçus:** Au total, 5787 alertes ont été reçues au Service d´Assistance Médical et d´Urgence (SAMU national) au cours de la période allant du 2 mars 2020 au 1^er^ mars 2021 avec un pic hebdomadaire de 291 alertes correspondant à la semaine 26.

**Test de dépistage de COVID-19:** entre le 2 mars 2020 et le 1^er^ mars 2021, 419545 tests de dépistage à la COVID-19 ont été réalisés au total dans les différents laboratoires. Parmi ces tests effectués, 34732 ont été positifs soit un taux de positivité de 8.3%. La majorité des cas positifs ayant été détectés par les laboratoires de l´Institut Pasteur de Dakar.

**Taux d´attaque:** il a été en moyenne à 208 pour 100.000 habitants, avec des extrêmes de 568 et 21 (Tableau 5).

**Tableau 5:** situation des taux d´attaque par région

Région	Population	Nombre de cas de COVID-19	Taux d´attaque / 100 000 hbts
Dakar	3 835 019	21774	568
Thiès	2162 831	4330	200
Diourbel	1 859 503	1665	90
Kaolack	1191 566	1531	128
Saint-Louis	1 091 740	1239	113
Louga	1 061 607	978	92
Fatick	900 791	738	82
Tambacounda	872 156	313	36
Kolda	821 998	379	46
Matam	732 866	364	50
Kaffrine	728 948	150	21
Ziguinchor	683 952	657	96
Sédhiou	572 099	175	31
Kédougou	190 513	439	230
**Total**	16 705 589	34 732	208

## Discussion

Après un an d´épidémie, la capacité de résilience du système de santé du Sénégal a été mise à rude épreuve. Malgré toutes les dispositions prises pendant la phase de préparation, les vagues épidémiques n´ont pu être évitées. Toutefois, elles ont été tant bien que mal contenues. Le Sénégal fait partie des premiers pays africains touchés par la COVID-19, le quatrième [10]. Le trafic aérien étant important avec l´Europe, le premier cas était importé, en provenance de la France. L´importation a été au début, la première voie de contamination en Afrique [11]. Pendant la première année, l´épidémie a évolué en deux vagues avec une deuxième vague plus intense, en nombre de cas, de cas graves et de décès. Pendant la première vague le minimum de cas journaliers notifiés était de 207, elle était passée à 462 pendant la seconde. Généralement, en Afrique la deuxième vague a été plus intense que la première [12]. L´apparition de la deuxième vague peut être considérée comme un revers de la médaille du succès du Sénégal dans la gestion de sa première vague [13,14]. Les populations ont pensé que l´épidémie était terminée et ont commencé à moins respecter les mesures barrières. L´évolution des alertes provenant de la population reçus par les services d´urgences a suivi celle la courbe épidémiologique. Avec 34 732 cas en une année, pour une population de plus de 16 millions d´habitants, soit une incidence de 0,21%, l´impact du COVID-19 n´a pas été aussi dramatique que ce que les prédictions le suggéraient. Concernant ce faible impact, plusieurs hypothèses ont été émises [15-17]. Toutefois au-delà des questions environnementales et de population, la rapidité de réaction ainsi que les leçons tirées des précédentes épidémies marquantes telles que Ébola en Afrique de l´Ouest en 2014, ont permis au Sénégal et à beaucoup de pays africains d´affiner leur capacité de préparation et de réponse face aux urgences de santé publique. La mise en place de centre des opérations d´urgence sanitaire, malgré tous les défis [18] a permis aux pays d´être mieux préparés face aux évènements de santé publique. Le centre des opérations d´urgence sanitaire du Sénégal est un des premiers fonctionnels sur le continent et en constitue un modèle [19]. Il a été créé en décembre 2014 et depuis, a travaillé dans le sens du renforcement de la capacité de préparation du pays.

La stratégie du Sénégal dans la lutte contre la COVID-19 a été dynamique, tenant compte du contexte local et des données scientifiques du moment. Très tôt, dans les 3 semaines après, alors que le nombre de cas n´atteignait pas dix, des mesures fortes ont été prises par les autorités allant dans le sens de la limitation des mouvements des populations : interdiction de manifestations publiques, fermeture des écoles et universités, restriction du transport interurbain. L´obligation du port de masque dans les lieux publics et les transports a été décidé le 7 avril 2020. Les actions prises très tôt pour éviter la transmission dans les transports en commun ont été notables, car étant le principal moyen de déplacement de la population en zone urbaine et interurbaine d´autant plus que les transports sont reconnus comme étant les lieux de contamination par prédilection [20]. La fermeture des frontières terrestres et aériennes a été également une décision importante qui a permis de réduire les cas importés. Elle impacte sur la progression de l´épidémie [21,22], même si l´Organisation mondiale de la Santé ne la recommande pas [23]. Près de 62,7% des cas sont localisés dans Dakar, la capitale, concentrant environ 23 % de la population. Dans la plupart des pays, la capitale constitue généralement l´épicentre de l´épidémie [24,25].

Le Sénégal ayant une population se caractérisant par sa jeunesse : un peu plus de la moitié de cette population (50.04%) a moins de 19 ans [8]; cela se perçoit sur la proportion de jeune touchée : 35,8% avaient moins de 35 ans. La moyenne d´âge des patients était de 47 ans avec des extrêmes de 7 mois et 101 ans. Le patient le plus âgé est malheureusement décédé des suites de sa maladie. Les personnes de plus de 60 ans atteintes représentent 23,9%. Nous notons de même une prédominance masculine, avec une proportion de 55,5% d´hommes et 44,5% de femmes, ceci est également retrouvé dans plusieurs études [26,27]. Les enfants, généralement peu touchés, [28] représentent 3,4% (moins de 14 ans). Les premiers cas ont d´abord été importés, ensuite des contacts de ces cas ont été infectés. Ceci a été le cas pour la plupart des pays africains qui n´ont pas pu éviter d´être touchés par cette pandémie [16, 29]. Le premier cas de transmission communautaire a été identifié le 19 mars 2020. Cette phase de transmission communautaire a intensifié la transmission dans tout le pays, créant par moment une certaine psychose dans la population. La crainte du « cas communautaire » poussait les populations à plus de respect des mesures barrières. Plus de 400 000 tests ont été réalisés par neuf laboratoires, avec un taux de positivité de 8,3%. L´institut Pasteur a été le premier laboratoire en mesure de poser le diagnostic, jusqu´au 30 mars. Il a eu a effectué près de 73,8% des tests, avec une décentralisation de leur laboratoire dans les régions périphériques. La multiplication des laboratoires agréés et la stratégie de décentralisation visaient à réduire les délais de rendu de résultats à moins de 48 heures. Cette stratégie de décentralisation du diagnostic est décisive dans la maitrise de la propagation de l´épidémie, elle permet aux équipes de terrain de pouvoir rapidement démarrer la prise en charge, permettant de rompre la chaine de transmission.

Le suivi des contacts a été très difficile avec l´évolution de l´épidémie. Il a été bien conduit pendant les six premiers mois, avec 38 612 contacts enregistrés pour 13 826 cas positifs, à la date du 2 septembre 2020, avec une proportion de suivi à 92%. Ce suivi a évolué à cause de la forte stigmatisation qui frappait les équipes de districts, avec des menaces sur leur intégrité physique. Le suivi physique a laissé place au suivi téléphonique, à distance. L´augmentation des cas dans la deuxième vague a entrainé une surcharge de travail des équipes de district. Ces équipes avaient en charge aussi la détection, l´isolement, les prélèvements et le transport des cas confirmés vers les centres de traitement en plus de leurs activités de routine. Le déficit en ressource humaine, malgré le renforcement des équipes par du personnel de la croix rouge a poussé les équipes à prioriser la prise en charge des cas au détriment du suivi des contacts. Le suivi des contacts est une activité clé dans la maitrise de la transmission des maladies infectieuses [30,31]. Il faut cependant noter la difficulté dans l´épidémie COVID-19 de procéder à un bon suivi des contacts lors de la phase de transmission communautaire, où le traçage des contacts est très difficile, du fait de mode de contamination. Les cas graves représentaient 2,8% des patients. Le taux de létalité était de 2,5%. Les décès étaient notés dans 3 endroits : les centres de traitement (88,4%), les structures de santé hors centre de traitement (5%) et les domiciles (6,6%). Dans les domiciles, il s´agissait de diagnostic post-mortem. La proportion importante de décès à domicile était grandement due à la stigmatisation qui entoure la maladie et la peur des populations de fréquenter les structures de soin. La moyenne d´âge des décédés était de 69 ans, avec une large majorité d´homme, les personnes de plus de 60 ans représentaient 73,9% des décès, ceci est également retrouvé dans la littérature [32]. Les patients de plus de 60 ans semblent plus être à risque [33,34]. Parmi les personnes décédées, 62% avaient au moins un diabète ou une hypertension artérielle, ces pathologies sont les comorbidités les plus retrouvées [35]. Les affections cardiovasculaires sont reconnues comme facteur de risque important [36]. Seuls 4,7% présentaient une pathologie respiratoire (bronchopneumopathie chronique obstructive ou asthme). Les personnes de moins de 35 ans décédées ne représentaient que 3,9%. Des décès ont été notés dans la littérature chez les enfants [37], dans notre série nous avons noté trois chez les moins de 14 ans, soit 0,3% des décès. Les capacités de prise en charge ont été développées tout en gardant comme ligne directrice la continuité de l´offre de soin pour les autres affections et l´accessibilité dans toutes les régions du pays. Le défi principal était de ne pas transformer nos hôpitaux en « hôpitaux COVID ». Le redéploiement du personnel de santé, la mobilisation d´étudiants en médecine en fin de cycle et des personnels retraités aptes ont permis de soutenir la résilience du système hospitalier.

## Conclusion

La pandémie COVID-19 continue d´être une grande menace pour le Sénégal, malgré les efforts déployés et les résultats obtenus. Une stratégie dynamique de gestion a été adoptée, tenant compte du contexte local, des moyens disponibles et des connaissances scientifiques. Les leçons apprises des différents événements de santé publique ont également contribué à renforcer les capacités de préparation et de gestion des crises sanitaires par le pays.

### Etat des connaissances sur le sujet

- *Bonne connaissance de l´épidémiologie COVID-19 en Europe, aux USA et Asie*.

### Contribution de notre étude à la connaissance

- *Avoir une meilleure connaissance du contexte COVID en Afrique ;*
- *Contribuer au forum scientifique sur COVID-19*.
